# Distribution of Melanin Pigmentation in 33 Organs of Thai Black-Bone Chickens (*Gallus gallus domesticus*)

**DOI:** 10.3390/ani10050777

**Published:** 2020-04-30

**Authors:** Korakot Nganvongpanit, Piyatida Kaewkumpai, Varankpicha Kochagul, Kidsadagon Pringproa, Veerasak Punyapornwithaya, Supamit Mekchay

**Affiliations:** 1 Department of Veterinary Biosciences and Public Health, Faculty of Veterinary Medicine, Chiang Mai University, Chiang Mai 50100, Thailand; piyatida.kk@gmail.com (P.K.); kidsadagon.p@cmu.ac.th (K.P.); 2 Excellence Center in Veterinary Bioscience, Chiang Mai University, Chiang Mai 50200, Thailand; 3 Veterinary Diagnostic Laboratory, Faculty of Veterinary Medicine, Chiang Mai University, Chiang Mai 50100, Thailand; chomchay.k@cmu.ac.th; 4 Department of Food Animal Clinic, Faculty of Veterinary Medicine, Chiang Mai University, Chiang Mai 50200, Thailand; veerasak.p@cmu.ac.th; 5 Department of Animal and Aquatic Sciences, Faculty of Agriculture, Chiang Mai University, Chiang Mai 50200, Thailand; supamit.m@cmu.ac.th

**Keywords:** accumulate, chicken, hyperpigmentation, melanosome, tissue

## Abstract

**Simple Summary:**

Consumers are selectively attracted to the black-bone chicken breed for the characteristic darkness that is observed in many of its organs. However, the degree of darkness in all organs of the black-bone chicken is still relatively unknown. The question of whether hyperpigmentation is present in every organ or just in some organs is an important one. Additionally, the question of whether a similar hyperpigmentation pattern exists in all animals is also of significant interest. Presently, no reports have yet attempted to explain the distribution and location of the melanin pigment that is present in Thai Royal black-bone chickens. The results of this study will help to provide valuable background knowledge with regard to the anatomy of the black-bone chickens. We found that gender does not have an effect on the hyperpigmentation of each organ in black-bone chickens. Organs and/or tissue samples taken from some of the chickens, such as the heart, kidney, and abdominal fat, did not reveal any degree of hyperpigmentation while the liver of all chickens did not display any degree of hyperpigmentation. Finally, most organs did reveal some degree of melanin pigmentation in all layers. This was true with the exception of certain layers of the collected tissue samples, such as in the tissue samples of the epithelium and the tunica mucosa in some organs, that did not display any degree of pigmentation.

**Abstract:**

The black-bone chicken (*Gallus gallus domesticus*) is a breed of chicken that is commonly found in Thailand. This breed is known for having a number of black colored organs. Consumers have been notably attracted to the black-bone chicken breed for the characteristic darkness that is observed in many of its organs. However, the degree of darkness in all organs of the black-bone chicken is still in question. Importantly, there have not yet been any published reports on the distribution of melanin pigment in the organs of the black-bone chicken. This research study aims to examine the distribution of the melanin pigment in 33 organs of the Thai black-bone chicken. Ten black-bone chickens (five male, five female) were included in this study. Thirty-two organs including the brain, spinal cord, sciatic nerve, larynx, trachea, syrinx, lungs, heart, pericardium, aorta, brachial vein, kidney, cloaca, oviduct, testis, gastrocnemius muscle, femur, tongue, esophagus, crop, proventriculus, gizzard, duodenum, jejunum, ileum, cecum, pancreas, liver, gall bladder, omentum, abdominal fat, spleen, and skin were examined in this study. Histological sections taken from tissue samples of each of these organs were studied. The findings revealed that the presence of the melanin pigment was not significantly different (*p* > 0.005) between male and female specimens. Notably, the liver was the only organ in which the melanin pigment had not accumulated. Consequently, there was not a uniform pattern of melanin pigment accumulation throughout the organs of the chickens. The melanin pigment was present in all of the tissue layers of most organs, while the melanin pigment was found in only specific layers of some of the organs. In conclusion, the distribution of melanin pigmentation in the organs of each of the animals in this study was found to be different. However, in some tissue samples, such as those obtained from the liver, no accumulation of the melanin pigment was observed.

## 1. Introduction

Thai black-bone chicken (*Gallus gallus domesticus*) is a common breed of chicken that has been traditionally raised in the rural areas of the north of Thailand. Notably, the Thai black-bone chicken is similar in appearance to the native chicken of this region. However, this chicken species possesses some differing characteristics from native chickens including having a number of body parts that appear black in color, such as the mouth, tongue, face, comb, wattle, legs, nails, and skin [1]. The Thai black-bone chicken is known to have originated from Mongolia. At present, there are a number of breeds of black-bone chickens being raised in Thailand, such as the Indonesian black-bone chicken, the Japanese black-bone chicken, the Pheasant chicken, the Australian black-bone chicken, the Phu-Phan black chicken, and the Hmong black-bone chicken. This species is known to be tolerant and adaptable to the weather and climate of the northern region of Thailand. Consequently, it has become very common in the northern region of Thailand [2]. The black-bone chicken is a chicken with special properties, many of which are attributable to its melanin pigmentation and canosine content [3]. Many people in the region are known to consume the black-bone chicken as a medicinal food because it is believed to be able to nourish the body. Thus, the black-bone chicken breed has come to be seen as economically valuable within the country. In particular, the melanin of this chicken is now being used in commercial applications in the fields of medicine, pharmacology, cosmetics, as well as in others [1,3,4,5].

Melanocytes are the main cell type present in avian species and mammals. This cell type produces the melanin pigment that is responsible for determining body color [6,7]. Basically, epidermal melanocytes in humans transfer mature melanosomes via the dorsolateral route to the adjacent keratinocytes by way of melanocyte dendrites [8]. However, in avian embryos, there are two major migration waves of neural crest (NC) cells present at the trunk level [9]. The first wave of NC cells migrates ventrally through the trunk between the neural tube and the somite. Theses NC cells give rise to glia and neurons. Twenty-four hours following ventral migration, the NC cells begin to move along a dorsolateral path between the somite and the ectoderm. NC cells ultimately invade the ectoderm where they are differentiated into melanocytes [10]. According to a previous study involving Silky Fowl chickens, the melanocytes present in the internal organs of stage III melanosomes (the intermediate phase of melanosome production associated with deposits of melanin during the production of matrix proteins) produce and maintain melanosomes inside the cells [11]. Moreover, pigmentation is known to occur in black-bone chickens via two different migration pathways: the dorsolateral and the ventral pathways [12]. However, in other breeds of chicken, such as the White Leghorn [13], normally pigmentation normally only migrates dorso-laterally.

Melanins are produced and stored in melanosomes within the melanocytes that are known to produce two chemically distinct types of melanin pigments, namely the eumelanin and the phaeomelanin [14,15]. Melanin is an indole-containing biopolymer that can protect tissues from ultraviolet radiation by shielding DNA against damage. Moreover, melanin also displays an antioxidant function [5,16], as well as anti-inflammatory [17] and immunoregulatory functions in innate immune responses [18]. The occurrence of hyperpigmentation among Silky Fowl chickens may have a close relationship with immune system development in the Silky Fowl chicken breed [19].

The most well-known breed of black-bone chicken is the Silky Fowl chicken, a native Chinese breed with hyperpigmentation in its tissue and organs, such as in the dermal layer of the skin, as well as the bones, muscles, pleura, trachea, blood vessels, abdominal lining, and connective tissue [20,21]. However, no reports have yet attempted to explain the distribution and location of the melanin pigment present in Thai Royal black-bone chickens. This study aims to describe the distribution, location, and accumulation of melanin pigment in 32 organs of the Thai Royal black-bone chicken. The results of this study will help to fulfill valuable background knowledge with regard to the anatomy of the black-bone chicken.

## 2. Materials and Methods

### 2.1. Samples

Ten adult Thai Royal black-bone chicken carcasses (five male, five female) were obtained from the Royal Project Center, Chiang Mai, Thailand. Specifically, 33 organs (Table 1) were observed to be black in color. The protocol for the use of carcasses in this study was approved of by the Animal Ethics Committee, Faculty of Veterinary Medicine, Chiang Mai University (License number S23/2561).

### 2.2. Histological Study

Tissue preparation technique was employed following that which was described in previous studies [22]. Briefly, tissues were fixed in 10% neutrally buffered formalin for 24 h. The specimens were cut into 1 mm pieces, placed in plastic cassettes, and then processed in 10% formalin for 1 h (two changes), 95% ethanol for 1 h (three changes), absolute isopropyl alcohol for 1 h (two changes), xylene for 1 h (two changes), and Paraplast for 1 h (three changes). The tissues were then embedded in paraffin and cut into 5 µm sections.

Sections were deparaffinized in xylene and rehydrated through a series of steps involving alcohol and water. Tissue sections were stained with Harris’s hematoxylin for 5 min and washed under running tap water for 5 min; differentiated in 1% acid alcohol (1% hydrochloric acid in 70% ethanol) for 5 s and washed under tap water for 5 min; dipped in saturated lithium carbonate solution for 5 s and washed under tap water for 5 min; and stained with 1% eosin Y for 3 min and washed under running tap water for 5 min. The sections were then dehydrated through a graded ethanol series, cleared in xylene, and mounted in Permount. Individual sections were examined in order to determine the distribution of the pigment using a compound light microscope (Olympus BX53, Tokyo, Japan).

In each organ of each chicken, the tissue was randomly cut in three places to produce three pieces. Additionally, each piece of tissue was used to prepare three slides. Each slide was representative of an image of the entire tissue area. Axio Vision 4.8.2 software (Carl Zeiss, Berlin, Germany) was used to record images in order to calculate the percentage of melanin pigment. The percentage of the melanin pigment was calculated from 100*(area of melanin pigment/area of all tissue) using image J software in the study of the accumulation of the melanin pigment in each organ.

### 2.3. Data and Statistical Analysis

Data on the macroscopic examination and pattern of the melanin pigment are presented using detailed descriptions. The fisher exact test was used to compare a proportion of the specimens as a hyperpigmentation between sexes. A *p*-value of <0.05 was considered statistically significant. ANOVA and *t*-test were used to compare the differences in mean values of the percentages for the accumulation of the melanin pigment among 33 organs and sexes. The mean values of the percentages for the accumulation of the melanin pigment in each organ are shown descriptively using a clustered heat map (a combination of a dendrogram and a heatmap) [23,24]. A dendrogram is a tree representing hieratical clustering, which is a method that combines data points into clusters, those clusters into larger clusters and so forth, creating a hierarchy [25,26]. Heatmaps visualize a data matrix by creating a rectangular grid that corresponds to rows and columns in the matrix, and the cells are colored according to their values in the data matrix. In this study, we colored the cells accorded to the percentage of melanin pigment, where the highest melanin pigment is represented by the color black and the lowest melanin pigment is presented in super light gray. All statistical analyses and the dendrogram generation were performed using R version 3.6.3 [27].

## 3. Results

According to the presence of the color black present in 33 organs of ten chickens, the liver was the only organ that did not appear black in color in all 10 chickens (Table 1). Notably, some organs and/or tissue samples did not appear to be black in color in all 10 chickens including the heart (6/10), kidney (9/10), and abdominal fat (5/10). However, the remaining 27 tissue samples of all 10 chickens appeared to be black in color.

**Figure 1:**
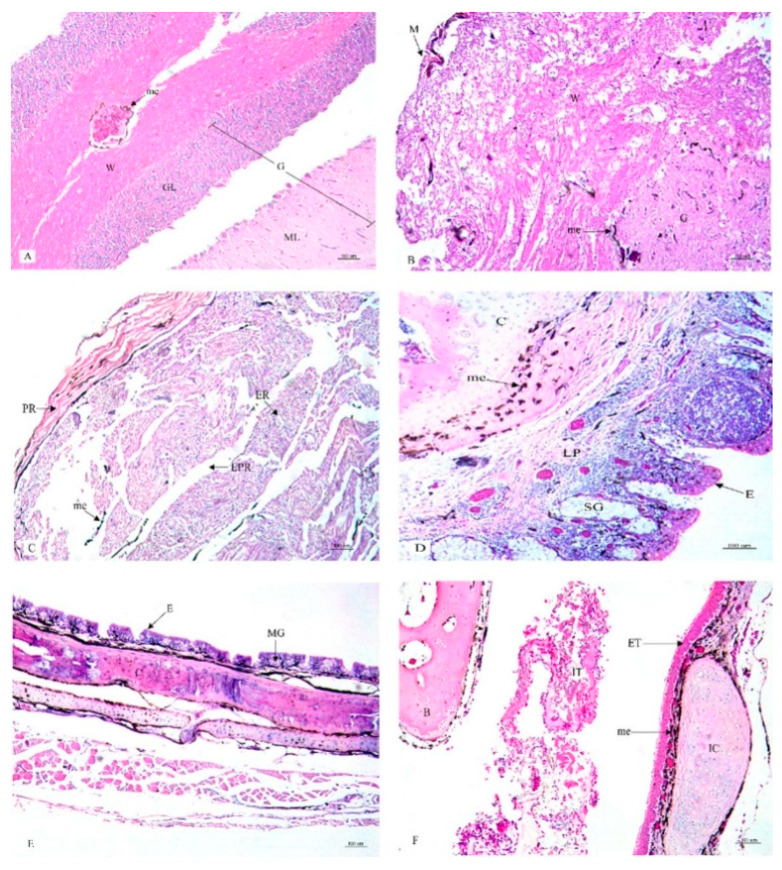
Histological location of melanin pigment in each organ; brain (**A**), spinal cord (**B**), sciatic nerve (**C**), larynx (**D**), trachea (**E**), syrinx (**F**). Abbreviations: M, meninges; G, gray matter; W, white matter; GL, granular layer; ML, molecular layer; PR, perinurium; ER, endonurium; EPR, epinurium; E, epithelium; LP, lamina propria; MG, mucous gland; C, cartilage; SG, seromucous gland; ET, external tympanic membrane; IT, internal tympanic membrane; IC, intermediate syringeal cartilage; B, bony pessulus;, me, melanin pigment. Low magnification, H&E stain.

**Figure 2:**
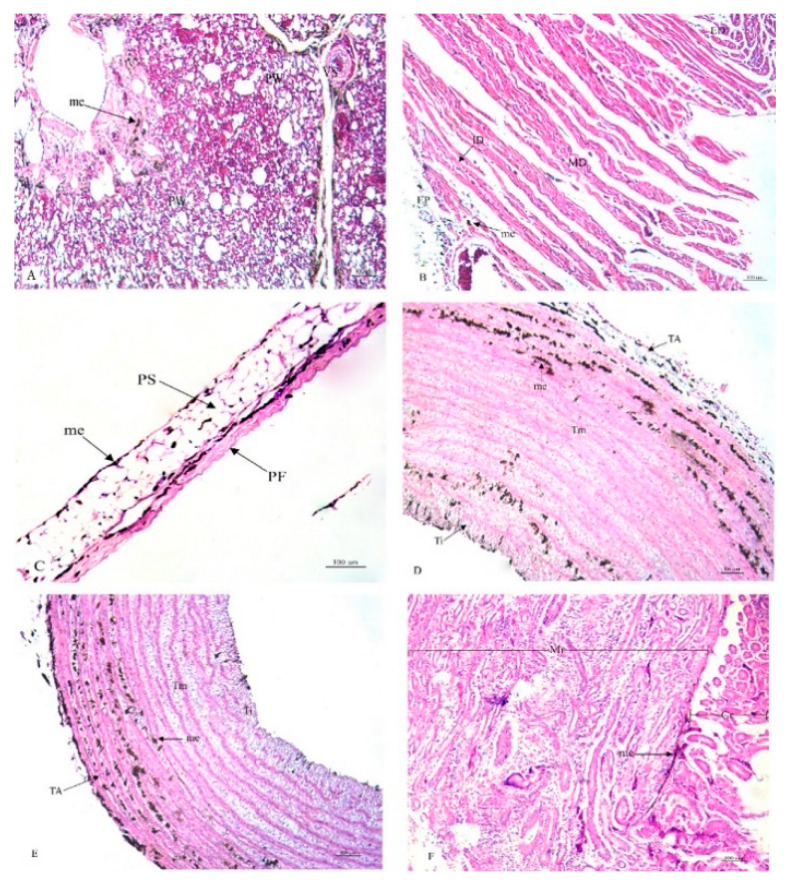
Histological location of melanin pigment in each organ; lung (**A**), heart (**B**), pericardium (**C**), aorta (**D**), brachial vein (**E**), kidney (**F**). Abbreviations: PW, parabronchial wall; VS, blood vessel; EP, epicardium; MD, myocardium; ED, endocardium; PF, fibrous pericardium; PS, serous pericardium; TA, tunica adventitia; Tm, tunica media; Ti, tunica interna; Cr, cortex; Mr, medulla; me, melanin pigment. Low magnification, H&E stain.

**Figure 3:**
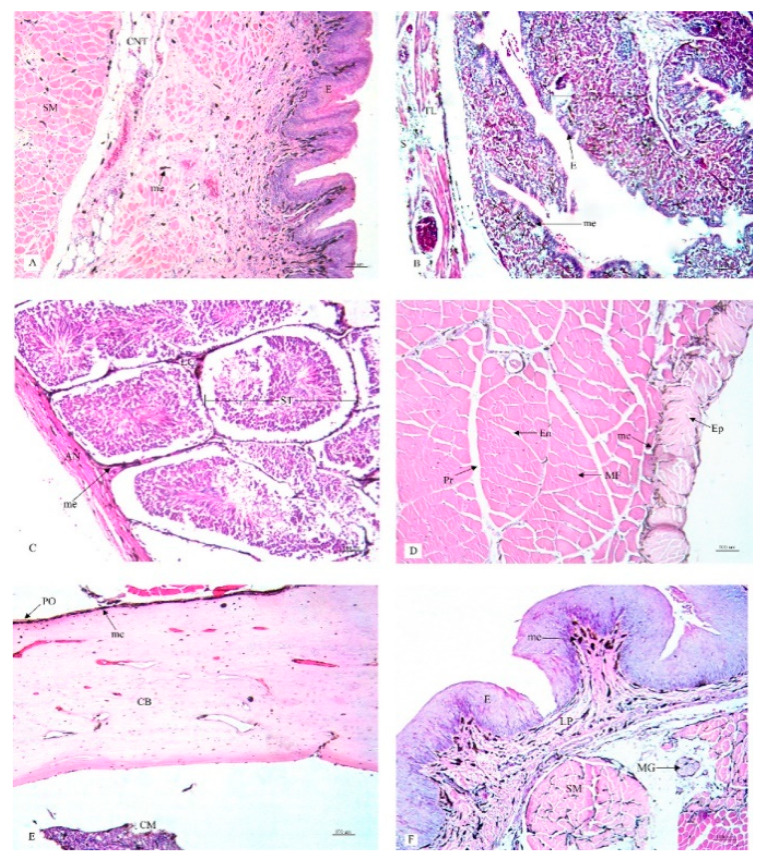
Histological location of melanin pigment in each organ; cloaca (**A**), oviduct (**B**), testis (**C**), gastrocnemius muscle (**D**), femur (**E**), tongue (**F**). Abbreviations: S, tunica serosa; TL, tunica muscularis; TS, tunica submucosa; TM, tunica mucosa; MG, mucous gland; E, epithelium; AN, tunica albuginia; LC, leydig cells; ST, seminiferous tubule; Ep, epimysium; Pr, perimysium; En, endomysium; MF, muscle fiber; PO, periosteum; CB, compact bone; CM, medullary cavity; E, epithelium; C, cartilage; me, melanin pigment. Low magnification, H&E stain.

**Figure 4:**
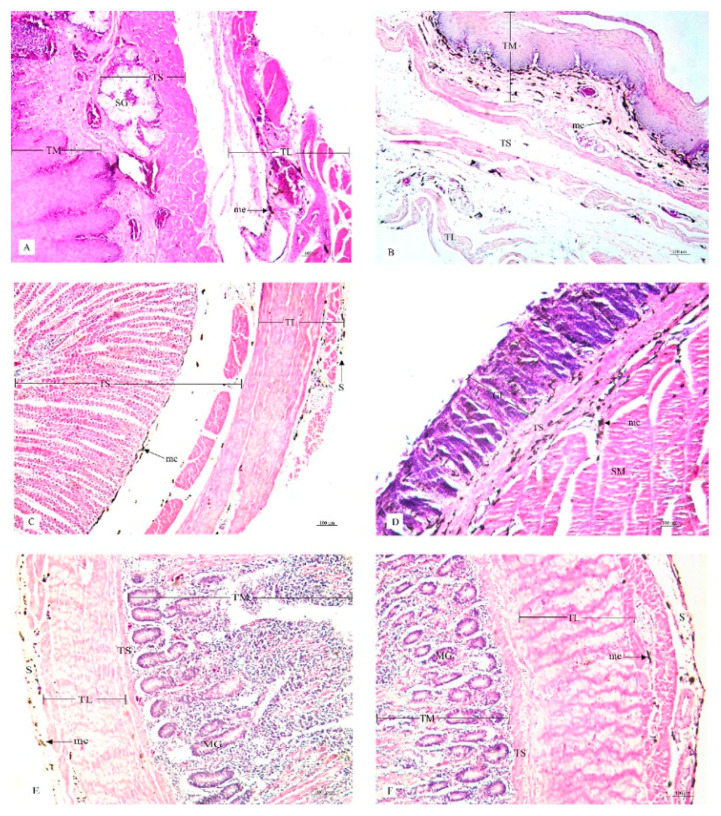
Histological location of melanin pigment in each organ; esophagus (**A**), crop (**B**), proventriculus (**C**), gizzard (**D**), duodenum (**E**), jejunum (**F**). Abbreviations: TL, tunica muscularis; TS, tunica submucosa; TM, tunica mucosa; SG, seromucous gland; S, tunica serosa; GL, granular layer; SM, muscular layer; MG, mucous gland; me, melanin pigment. Low magnification, H&E stain.

**Figure 5:**
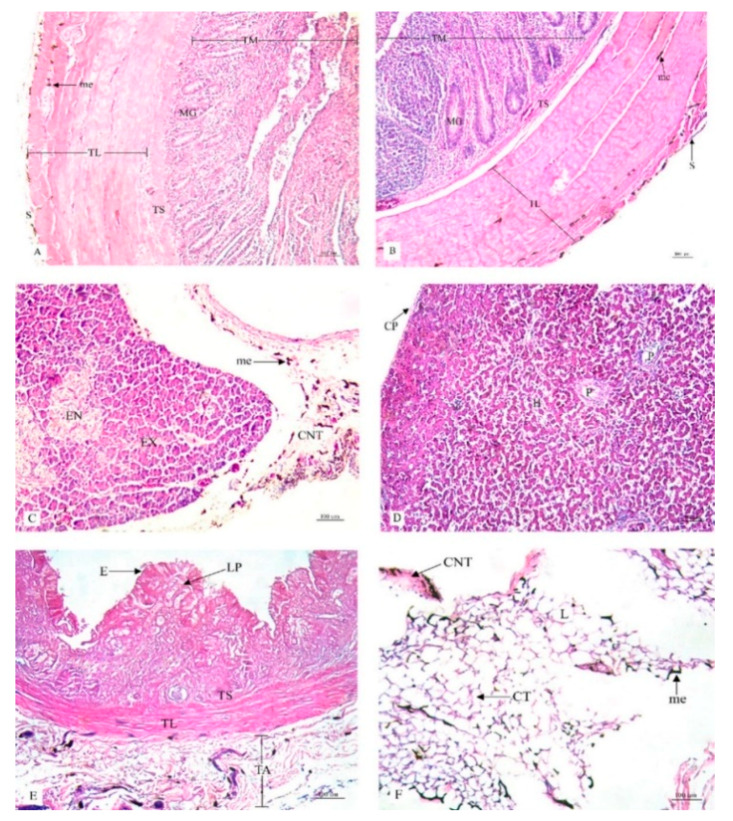
Histological location of melanin pigment in each organ; ileum (**A**), cecum (**B**), pancreas (**C**), liver (**D**), gall bladder (**E**), omentum (**F**). Abbreviations: S, tunica serosa; TL, tunica muscularis; TS, tunica submucosa; TM, tunica mucosa; MG, mucous gland; EX, exocrine; EN, endocrine; CNT, connective tissue; CP, capsule; H, hepatic lobule; P, portal tract; TA, tunica adventitia; E, epithelium; LP, lamina propria; CT, cytoplasm; L, lipid droplet; me, melanin pigment. Low magnification, H&E stain.

**Figure 6:**
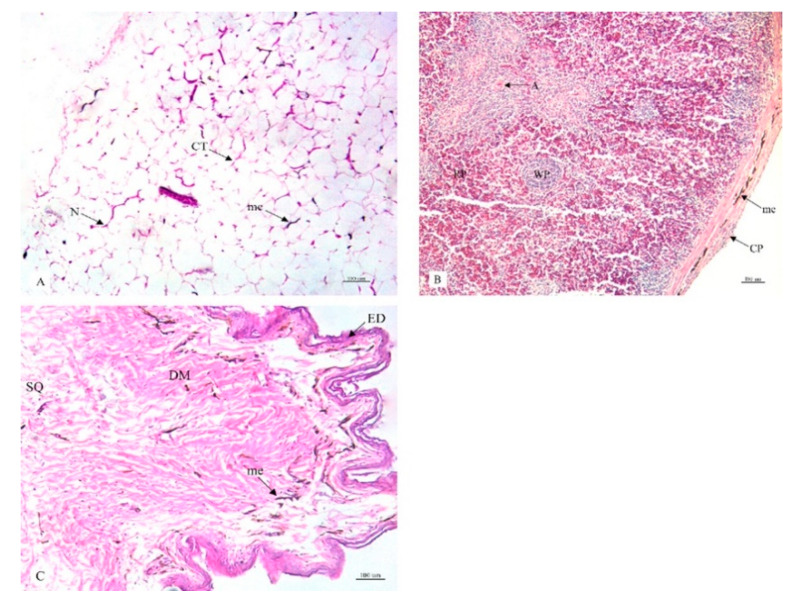
Histological location of melanin pigment in each organ; abdominal fat (**A**), spleen (**B**), skin (**C**). Abbreviations: CT, cytoplasm; N, nucleus; CP, capsule; RP, red pulp; WP, white pulp; A, central artery; ED, epidermis; DM, dermis; SQ, subcutis; me, melanin pigment. Low magnification, H&E stain.

This research study included a histological examination of the location and distribution of the melanin pigment. Primarily, we found that the melanin pigment was dispersed throughout most of the internal organs of the black-bone chickens in this study except for the liver, which did not reveal any melanin pigmentation. In addition, no significant differences (*p* > 0.05) were observed in all organs between male and female specimens in this study (Table 1). The location and distribution of the melanin pigment in each organ of the chickens are presented in the following description: Figure 1; the brain, spinal cord, sciatic nerve, larynx, trachea, and syrinx, Figure 2; lung, heart, pericardium, aorta, brachial vein, and kidney, Figure 3; cloaca, oviduct, testis, gastrocnemius muscle, femur, and tongue, Figure 4; esophagus, crop, proventriculus, gizzard, duodenum, and jejunum, Figure 5; ileum, cecum, pancreas, liver, gall bladder, and omentum, Figure 6; abdominal fat, spleen, and skin. Additionally, in some organs, the melanin pigment was found to have accumulated in all layers of the tissue samples. However, the melanin pigment was only observed in some layers of certain tissue samples as follows: tracheal (Figure 1), cloaca (Figure 3), oviduct (Figure 3) while no melanin pigment was found in the epithelium layer. In tissue samples of the esophagus (Figure 4), the melanin pigment was only found in the tunica serosa, crop (Figure 4), ilium (Figure 4), and cecum (Figure 4), while no melanin pigment was found in the tunica mucosa jejunum and in the tunica submucosa. In tissue samples of the pancreas (Figure 5), the melanin pigment was only found in the connective tissue, and in the spleen the melanin pigment was found only in the capsule (Figure 6).

**Figure 7:**
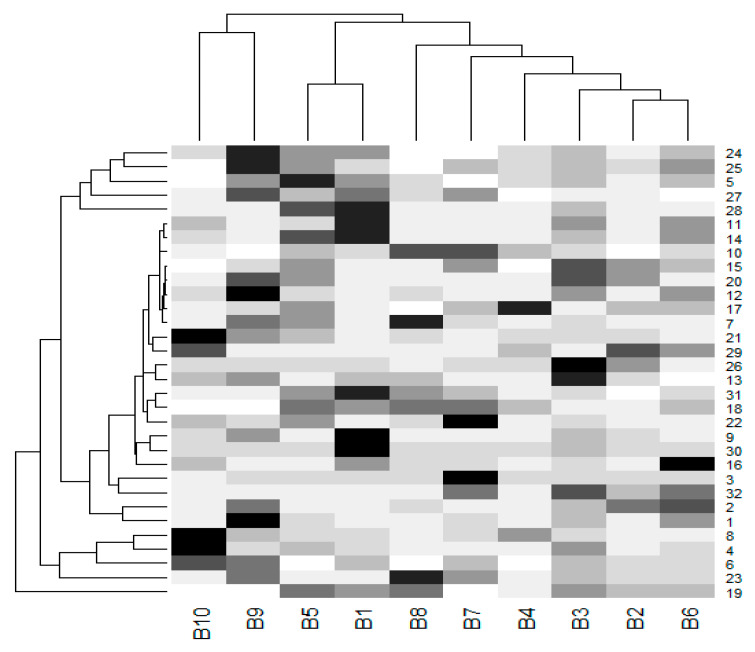
A dendrogram was prepared from the percentages of melanin pigment (mean values) found in 32 organs of each chicken. The cells are colored according to the percentage of melanin pigment. The highest melanin pigment is represented by the color black, while the lowest melanin pigment is presented in super light gray. A tree on the column represents a clustered group of chickens, while a tree on the row represents a clustered group of organs. (B-1-10, B1-5 were male subjects and B6-10 were female subjects). The liver, beak, comb, wattle, scale, and feathers were not included in this dendrogram. (1 = brain, 2 = spinal cord, 3 = sciatic nerve, 4 = larynx, 5 = trachea, 6 = syrinx, 7 = lung, 8 = heart, 9 = pericardium, 10 = aorta, 11 = brachial vein, 12 = kidney, 13 = cloaca, 14 = oviduct, 15 = testis, 16 = gastrocnemius muscle, 17 = femur, 18 = tongue, 19 = esophagus, 20 = crop, 21 = proventriculus, 22 = gizzard, 23 = duodenum, 24 = jejunum, 25 = ileum, 26 = cecum, 27 = pancreas, 28 = gall bladder, 29 = omentum, 30 = abdominal fat, 31 = spleen, 32 = skin).

Due to the small sample size (*n* = 10), the power of the test was too small to detect any differences in the mean values between genders using *t*-test or ANOVA. Moreover, the violation of normality and the homogeneity of variances are recognized as common problems. In addition, a comparison of the mean values of the percentages for the accumulation of the melanin pigment among 33 organs was unable to be conducted due to the high number of pairs that needed to be tested (number of pairs = 528) and the effects of adjustment method was employed to control the type 1 error (e.g., Tukey’s test). The mean values of the differing percentages of the melanin pigment are presented with the use of a dendrogram (Figure 7). The tree on the row could represent the clustering of only two main groups, esophagus and the 31 other organs. It was revealed that variations in the accumulation of the melanin pigment in all organs was not uniform. Additionally, the tree on the column could represent clustering into two main groups with two female subjects and the eight other chickens (three female and five male). It was determined that gender did not have an effect on the overall variations in the pattern of the melanin pigment.

## 4. Discussion

This research study was the first of its kind to identify the distribution of the melanin pigment in the organs of black-bone chickens. The highlights of this study include the following;

1. The liver was only one organ in which the melanin pigment was not found to have accumulated.
2. A uniform pattern of melanin pigment distribution was not observed among all organs of the chickens.
3. Gender had no influence on the presence of the melanin pigment.
4. The melanin pigment was found to be present in all tissue layers of most organs, while in some organs the melanin pigment was only found in specific layers.

It has been well established that melanoblasts in birds are known to migrate mainly via the dorsolateral and ventral routes originating from the neural crest [12,21]. This can be the cause of hyperpigmentation that is observed in many tissues and organs such as in the dermal layer of the skin, bone, muscle, pleura, trachea, blood vessels, abdominal lining, and connective tissue [19,20]. In a previous study [19], it has been shown that the melanin pigment accumulated in many internal organs of the black-bone chicken (Silky fowls) including the skin, connective tissues (periosteum, mesentery, epicardium, meninx, and peritoneum), skeletal muscle (breast muscle and leg muscle), respiratory tract (trachea and lung), immune organs (spleen, thymus, and bursa of Fabricius), intestines, and reproductive organs (oviduct, ovary, vas deferens, and testis). However, hyperpigmentation was not observed in some organs, such as the liver, pancreas, pituitary gland, and adrenal gland [19]. This outcome was similar to that of our study which found that 32 of 33 organs revealed the presence of a degree of hyperpigmentation except for the liver. Additionally, hyperpigmentation was found in the pancreas. Taken together, it can be concluded that not all organs of the black-bone chicken revealed melanin pigment accumulation, while certain organs and the nerves did display pigmentation including the liver, pituitary gland, and adrenal gland. However, we cannot explain the reason why these organs did not reveal any degree of hyperpigmentation. Importantly, embryological or developmental studies must be conducted for a clear understanding of this process.

The advantages of this study are that we have shown that each chicken possesses a different number of organs revealing melanin pigmentation. If scientists require black-bone chicken samples for the purposes of breeding or the study of genetics, they should select chickens who display a black color in most of their organs. Notably, some studies require that samples should possess extreme differences between two phenotypes [28]. This means that we need to include black-bone chickens that have mostly black colored organs in future research studies focusing on genetic selection or breeding. Additionally, most consumers prefer black-bone chickens that have all dark or black colored organs.

Our study has clearly shown that the layer-by-layer accumulation of the melanin pigment occurs in each tissue. Most tissue samples revealed melanin pigment accumulation in all layers; however, in some tissue samples, the melanin pigment was only found in specific layers. Conclusively, the melanin pigment is ubiquitous in various tissue layers and organs. However, the melanin pigment did not accumulate in the epithelial layer of the trachea, cloaca, oviduct, and gall bladder. Moreover, in the tunica mucosa of the gastrointestinal tract, i.e., esophagus, crop, jejunum, ileum, and cecum, melanin pigmentation was not observed. The epithelial layer and the tunica mucosa were located in the inner layer of the lumen of the organs; therefore, function was dependent upon the protection, absorption, and resorption processes of these organs [29,30]. The cell linings of most of these layers were unique and shared almost the same cell type. For example, the epithelium of the trachea was pseudostratified ciliated columnar with goblet cells, the gall bladder was columnar epithelium, the esophagus was stratified squamous epithelium or lined with epithelium columnar cells in the small intestinal and cecum regions [31]. The other layers were comprised of many cell types [31] for which the melanocytes could still remain within. In a previous study involving Silky fowls, it was also reported that the majority of the melanocytes were mainly present near blood vessels and in the muscle layer and outer membrane of the intestines [19].

Importantly, we have identified some limitations in this study that need to be addressed. First, our study did not examine the bonny tissue because of the occurrence of certain technical errors. However, previous studies had reported on the histological study of bones taken from black-bone chickens (Silky fowls). Melanin pigment levels were higher in accumulation in the periosteum, while lesser amounts were found to be present in the superficial layer of the compact bone [32]. With regard to other limitations of our study, we did not have success in utilizing an immunohistochemistry technique in staining the melanocyte. Consequently, we could not confirm the presence of melanocytes in some layers of the tissues that did not reveal melanin pigmentation.

## 5. Conclusions

This study reported on the anatomical differences in the hyperpigmentation of 33 organs of the black-bone chicken. Notably, the reported findings of this study can be used to fill the present gaps with regard to the anatomical knowledge of this issue. Importantly, gender did not have an effect on the hyperpigmentation of each organ in black-bone chickens. Some organs and/or tissue samples taken from some of the chickens, such as the heart, kidney, and abdominal fat, did not reveal any degree of hyperpigmentation, while the liver of all chickens did not display any hyperpigmentation. Finally, most organs revealed some degree of melanin pigmentation in all layers with the exception of some layers of tissue samples such as the epithelium and the tunica mucosa in some organs.

## Figures and Tables

**Table 1:** Detection of melanin pigment present in each layer of all tissue samples and comparisons between male and female subjects.

System	Organs	Layers	Male	Female
Nervous system	Brain	Meninges	5/5	5/5
		Gray matter	4/5	4/5
		White matter	0/5	2/5
	Spinal cord	Meninges	3/3	3/3
		Gray matter	3/3	3/3
		White matter	2/3	2/3
	Sciatic nerve	Epineurium	5/5	4/4
		Perineurium	5/5	4/4
		Endoneurium	5/5	4/4
Respiratory system	Larynx	Epithelium	0/5	2/5
		Lamina propria	5/5	5/5
		Elastic cartilage	5/5	5/5
	Trachea	Hyaline cartilage	3/3	4/4
		Epithelium	0/3	0/4
		Mucous gland	3/3	4/4
	Syrinx	External tympanic membrane	2/2	3/3
		Internal tympanic membrane	2/2	3/3
		Intermediate syringeal cartilage	2/2	3/3
		Bony pessulus	2/2	3/3
	Lung	Parabronchial wall	5/5	5/5
		Blood vessel	5/5	5/5
Circulatory system	Heart	Epicardium	1/5	3/5
		Myocardium	1/5	1/5
		Endocardium	1/5	0/5
	Pericardium	Fibrous pericardium	4/4	3/3
		Serous pericardium	4/4	3/3
	Aorta	Tunica adventitia	4/4	4/4
		Tunica media	4/4	4/4
		Tunica intima	4/4	4/4
	Brachial vein	Tunica adventitia	3/3	5/5
		Tunica media	3/3	5/5
		Tunica intima	0/3	3/5
Urinary system	Kidney	Capsule	2/5	1/5
		Cortex	1/5	0/5
		Medulla	4/5	2/5
	Cloaca	Epithelium	0/4	0/4
		Connective tissue	4/4	4/4
		Muscular layer	4/4	4/4
Reproductive system	Oviduct	Tunica serosa	0/0	3/3
		Tunica muscularis	0/0	3/3
		Tunica mucosa	0/0	3/3
		Epithelium	0/0	0/3
	Testis	Tunica albuginia	4/4	0/0
		Leydig cells	4/4	0/0
		Smooth muscle	4/4	0/0
		Sperm	0/0	0/0
Skeleton system	Gastrocnemius muscle	Epimysium	5/5	5/5
		Perimysium	5/5	4/5
		Endomysium	5/5	5/5
	Femur	Periosteum	4/4	5/5
		Compact bone	4/4	5/5
		Medullary cavity	2/4	3/5
Digestive system	Tongue	Epithelium	4/4	5/5
		Lamina propria	4/4	5/5
		Muscular layer	4/4	5/5
	Esophagus	Tunica serosa	5/5	4/4
		Tunica muscularis	0/5	0/4
		Tunica submucosa	0/5	0/4
		Tunica mucosa	0/5	0/4
	Crop	Tunica adventitia	5/5	5/5
		Tunica muscularis	5/5	5/5
		Tunica submucosa	5/5	5/5
		Tunica mucosa	0/5	0/5
	Proventriculus	Tunica serosa	5/5	5/5
		Tunica muscularis	5/5	5/5
		Tunica submucosa	5/5	5/5
		Tunica mucosa	5/5	5/5
	Gizzard	Connective tissue	3/5	5/5
		Muscular layer	4/5	5/5
		Tunica submucosa	2/5	2/5
		Gland in mucous membrane	1/5	1/5
	Duodenum	Tunica serosa	5/5	5/5
		Tunica muscularis	1/5	2/5
		Tunica submucosa	0/5	1/5
		Tunica mucosa	0/5	1/5
	Jejunum	Tunica serosa	5/5	4/4
		Tunica muscularis	2/5	2/4
		Tunica submucosa	0/5	0/5
		Tunica mucosa	0/5	0/5
	Ileum	Tunica serosa	5/5	5/5
		Outer longitudinal muscle	4/5	5/5
		Inner circular muscle	2/5	3/5
		Tunica submucosa	1/5	1/5
		Tunica mucosa	0/5	0/5
	Cecum	Tunica serosa	5/5	5/5
		Tunica muscularis	5/5	5/5
		Tunica submucosa	2/5	3/5
		Tunica mucosa	0/5	0/5
	Pancreas	Exocrine	0/5	0/5
		Endocrine	0/5	0/5
		Connective tissue	5/5	5/5
	Liver	Capsule	0/5	0/5
		Hepatic lobules	0/5	0/5
		Portal tracts	0/5	0/5
	Gall bladder	Tunica adventitia	3/3	1/1
		Tunica muscularis	1/3	0/1
		Tunica submucosa	0/3	0/1
		Lamina propria	0/3	0/1
		Epithelium	0/3	0/1
	Omentum	Connective tissue	5/5	5/5
		Cytoplasm	5/5	5/5
	Abdominal fat	Cytoplasm	0/0	3/3
Lymphatic system	Spleen	Capsule	5/5	4/5
		Red pulp	0/5	0/5
		White pulp	0/5	0/5
		Central artery	0/5	0/5
Integumentary System	Skin	Epidermis	0/5	1/5
		Dermis	5/5	5/5
		Subcutis	5/5	5/5

Note: 1—The number of samples found/number of samples observed in some tissues or layers were lower than five as a result of errors that occurred in the tissue preparation process. 2—Fisher’s exact test was used to compare the proportion of the specimen for each organ layer (same row) between male and female subjects. There were no statistical differences (*p* > 0.05) observed in the 108 layers of 33 organs.
